# Total Extracts of *Abelmoschus manihot* L. Attenuates Adriamycin-Induced Renal Tubule Injury via Suppression of ROS-ERK1/2-Mediated NLRP3 Inflammasome Activation

**DOI:** 10.3389/fphar.2019.00567

**Published:** 2019-05-28

**Authors:** Wei Li, Weiming He, Ping Xia, Wei Sun, Ming Shi, Yao Zhou, Weiwei Zhu, Lu Zhang, Buhui Liu, Jingjing Zhu, Yiye Zhu, Enchao Zhou, Minjie Sun, Kun Gao

**Affiliations:** ^1^Affiliated Hospital of Nanjing University of Chinese Medicine, Division of Nephrology, Jiangsu Province Hospital of Traditional Chinese Medicine, Nanjing, China; ^2^Division of Gerontology, The Third Affiliated Hospital of Nanjing University of Chinese Medicine, Nanjing, China; ^3^Department of Pathophysiology, Xuzhou Medical University, Xuzhou, China; ^4^Department of Pharmaceutics, China Pharmaceutical University, Nanjing, China

**Keywords:** *Abelmoschus manihot* L., total extract, renal tubular cell, Adriamycin nephropathy, oxidative stress, ERK1/2, NLRP3 inflammasome

## Abstract

*Abelmoschus manihot* (L.) Medik. (Malvaceae) is a herb used in traditional Chinese medicine to treat some kidney diseases. To date, the detailed mechanisms by which *A. manihot* improves some kinds of renal disease are not fully understood. In this study, we established Adriamycin-induced NRK-52E cells, the normal rat kidney epithelial cell line, injury, and Sprague-Dawley rats with Adriamycin-induced nephropathy to evaluate the role and mechanisms of total extracts of *A. manihot* flower (TEA) both *in vitro* and *in vivo*. We found that TEA ameliorated Adriamycin-induced cellular morphological changes, cell viability, and apoptosis through the suppression of protein oxidation and ERK1/2 signaling. However, this anti-oxidative stress role of TEA was independent of ROS inhibition. Adriamycin activated ERK1/2 signaling followed by activation of NLRP3 inflammasomes. TEA suppressed NLRP3 inflammasomes via inhibition of ERK1/2 signal transduction; decreased proteinuria and attenuated renal tubule lesions; and inhibited the expression of NLRP3 in tubules in rats with Adriamycin nephropathy. Collectively, TEA protects renal tubular cells against Adriamycin-induced tubule injury via inhibition of ROS-ERK1/2-NLRP3 inflammasomes.

## Introduction

Renal tubular cells constitute most of the mass of the kidney and maintain normal renal structure and function. Tubular cells exhibit a variety of degenerative changes or undergo acute reversible or irreversible damage in response to numerous stimuli. Tubular lesions and recovery play crucial roles in epithelial-to-mesenchymal transition, renal fibrosis, acute kidney injury to chronic kidney disease (CKD) transition, and the progression of kidney disease (Venkatachalam et al., 2015). The extent of renal dysfunction is closely associated with changes in glomerular and tubulointerstitial injury. As a final common pathway, tubular injury is a major determinant in the progression of kidney disease (Nath, 1992). Thus, it is clinically significant to fully understand the tubule and preservation of the structure and function of renal tubular epithelial cells may provide a novel therapy for slowing the progression of kidney disease. However, to date, efforts aimed at the preservation of renal tubular cells via pharmacological interventions have not yet achieved clinical significance. Therefore, herbs associated with traditional Chinese medicine are perceived to be a cost-efficient alternative.

Traditional Chinese medicine has developed over the last 2,500 years. Ancient people found many ways to treat edema and hematuria as well as symptoms of kidney disease. Chinese herbs have been proven to be an effective method of treating some kinds of kidney disease both in ancient and modern times. The flower of *Abelmoschus manihot* (L.) Medik. (Malvaceae) – its Chinese name is *Huang Shu Kui Hua* – is used for the treatment of some kidney diseases (Yu et al., 1995; Chen et al., 2016). The chemical constituents in the plant are mainly flavonoids, organic acids, steroids and volatile compounds. The compounds were isolated and purified by chromatographic techniques and their structures were identified on the basis of physicochemical properties and spectral data. Seventeen compounds were isolated and identified as quercetin, hyperoside, cannabiscitrin, quercetin-3′-glucoside, 8-(2″-pyrrolidinone-5-yl)-quercetin, myricetin, floramanoside F, isomyricitrin, dihydromyricetin, rutin, 3-*O*-kaempferol-3-*O*-acetyl-6-*O*-(*p*-coumaroyl)- α-D-glucopyranoside, adenosine, 5′-deoxy-5′-methylsulphinyl adenosine, uracil, nicotinamide, (*E*)-9- octadecenoic acid, and gallic acid (Yang et al., 2017). Five flavonoids, hyperoside, myricetin, quercetin, isoquercitrin, and rutin, have been determined to be the major pharmacologically bioactive components via high-performance liquid chromatography (HPLC) that simultaneously quantifies the flavonoid compounds of *A. manihot* L. flower (Lai et al., 2009). Huang Kui Capsule, the commercial name of a pharmaceutical preparation of the extract of *A. manihot* flower, acquired approval from the State Food and Drug Administration (SFDA) as a class III drug for treating chronic glomerulonephritis many years ago in China (Song and Lian, 2005; Zhang and Qu, 2010). A pharmacological study found that the effects of *A. manihot* might be associated with the inhibition of immune reactions and inflammation, amelioration of kidney fibrosis, anticoagulant effects, and so on (Chen et al., 2012). Meanwhile, Zhou et al. (2012) reported the proteinuria-lowering effects of *A. manihot* via the protection of podocytes. In our previous study, total extracts of *A. manihot* flower (TEA) was able to improve proteinuria in rats with Adriamycin nephropathy (AN) (Tu et al., 2013; Mao et al., 2015). A prospective multicenter randomized controlled clinical trial has confirmed that Huang Kui Capsule reduces proteinuria more effectively than losartan in patients with primary glomerular disease diagnosed by renal biopsy (Zhang et al., 2014). The mechanism underlying the role of *A. manihot* on CKD is not yet fully understood. Proteinuria is the result of both glomerular injury and tubular impairment (Gorriz and Martinez-Castelao, 2012). Although previous reports have documented the role of *A. manihot* in podocytes, questions have arisen as to whether *A. manihot* protects renal tubular epithelial cells.

Adriamycin nephropathy is a rodent model of kidney disease and is characterized by proteinuria and loss of renal function. It has been studied extensively and has enabled a greater understanding of the processes underlying the progression of chronic proteinuric renal disease (Bertani et al., 1982; Okuda et al., 1986). Adriamycin induces both glomerular and tubulointerstitial injury (Javaid et al., 2001). Oxidative stress is considered to be the most important mechanism underlying the cytotoxicity of Adriamycin. Over production of reactive oxygen species (ROS) is considered as the most important mechanism underlying the cytotoxicity of Adriamycin. It has been reported that Adriamycin can activate inflammasome through ROS (Nicholas et al., 2011; Sauter et al., 2011). From both clinical and experimental data, nucleotide-binding oligomerization domain, leucine-rich repeat, and pyrin domain containing-3 (NLRP3) inflammasomes have recently been documented to be involved in the pathogenesis of CKD and acute kidney injury. However, the detailed mechanisms are not fully understood in kidney. Since *A. manihot* has multiple pharmacological roles in kidney disease, increasing attention is being paid to its mechanisms in the kidney. In this study, we employed Adriamycin-induced injury in NRK-52E, a rat renal proximal tubular cell line, to determine whether and how TEA protects renal tubular cells *in vitro.* Meanwhile, Adriamycin-induced nephropathy in rats was established to confirm the role and mechanism of TEA *in vivo*.

## Materials and Methods

### Preparation of *A. manihot* Extract

TEA is a single plant drug extracted from the dry corolla of *A. manihot*. TEA was extracted by the Department of Drug Preparation of the Affiliated Hospital of Nanjing University of Chinese Medicine. Briefly, 0.5 kg of raw *A. manihot* flowers was immersed in 8 L 75% ethanol for 1 h and then heated to 90°C for 1 h to achieve alcohol extraction as ambrette fluid extract. After filtration, the extract was evaporated to produce a dry extract powder under vacuum at 60°C. The dried residue was dissolved in water for use in the experiments. The quality of TEA was examined with fingerprint analysis via HPLC.

### High Performance Liquid Chromatograph Analysis

A Waters e2695 HPLC (on-line degasser, quaternary pump, automatic sampler, and UV detector), with a Kh-500de ultrasonic cleaner (Kunshan Hechuang Ultrasonic Instrument Co., Ltd.), Sartorius CPA225D electronic balance (Nanjing Yimanelli Instrument and Equipment Co., Ltd.), and a C18 (250 mm, 4.6 mm, 5 m) chromatographic column were used in this study. Rutin (lot no. C10275778), hyperoside (lot no. C10180684), myricetin (lot no. C10079514), and quercetin (lot no. C10298448) were purchased from Shanghai Macklin Biotechnology Co., Ltd. Isoquercitrin (lot no. P25J9F65872) was purchased from Shanghai Yuanye Biotechnology Co., Ltd.

Single reference sample solutions of different volumes were measured, mixed, and diluted to produce mixed reference sample solutions containing five components. The concentration of each component was as follows: Rutin, 0.026 mg/mL; hyperoside, 0.056 mg/mL; myricetin, 0.154 mg/mL; quercetin, 0.020 mg/mL; and Isoquercitrin, 0.034 mg/mL. The sample (TEA) was diluted 16 times with distilled water and filtered through a 0.45-μm membrane filter.

The chromatographic method was as follows: we used a Dubhe C18 (250 mm, 4.6 mm, 5 m) chromatographic column, with a mobile phase of acetonitrile (A) -0.05% formic acid aqueous solution (B), a gradient elution of: (0–8 min, 15% A, 17% A; 8–45 min, 17% A, 30%A; 45–55 min, 30% A, 80%A; 55–56 min, 80% A, 95% A; 56–57 min, 95% A, 15% A; 57–60 min, 15% A), a velocity of 1.0 mL/min, and a detection wavelength of 360 nm, column temperature 35°C. The sample quantity was 10 μL.

**FIGURE 1:**
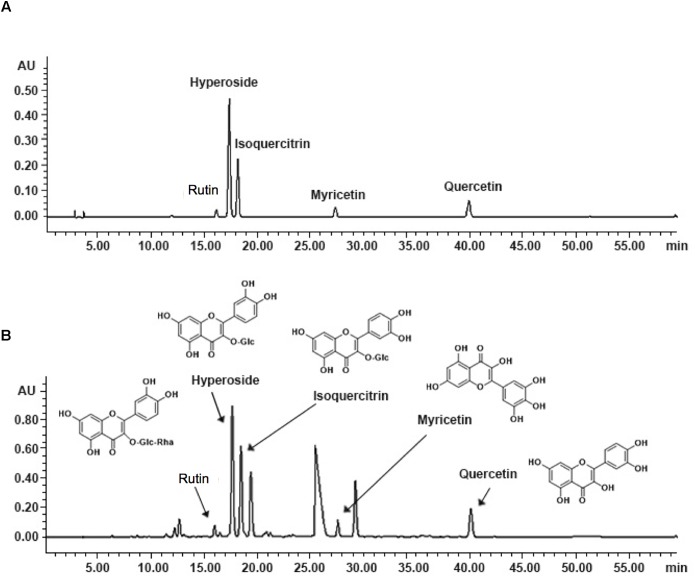
Fingerprint analysis of TEA by HPLC. **(A)** Chromatograms of mixed standards. **(B)** TEA samples.

As shown in Figure 1, the five known components of TEA include rutin (C_27_H_30_O_16_; CAS: 153-18-4), hyperoside (C_21_H_20_O_12_; CAS: 482-36-0), isoquercitrin (C_21_H_20_O_12_; CAS: 482-35-9), myricetin (C_15_H_10_O_8_; CAS: 529-44-2), and quercetin (C_15_H_10_O_7_; CAS: 117-39-5). The concentrations of hyperoside in different preparations were measured for quality control according to the Chinese Pharmacopoeia 2010.

### Cells

Normal rat tubular proximal epithelial cell lines, NRK-52E (from the University of Yamanashi, Japan), were maintained in Dulbecco’s modified Eagle’s medium/F-12 (DMEM/F12) supplemented with 100 U/mL penicillin G, 100 mg/mL streptomycin, 0.25 mg/mL amphotericin B, and 5% fetal bovine serum (FBS). Cells were incubated at 37°C in 5% CO_2_. All experiments were performed after cells were seeded in medium containing 1% FBS for 24 h.

### Animals

Male Sprague-Dawley rats (*n* = 30, 12 weeks old) weighing 230–250 g were purchased from the Animal Center of Yangzhou University (Yangzhou, China). After one week of adaptive feeding, the rats were randomly divided into three groups (each *n* = 10): normal, Adriamycin model, and TEA. The AN rat model was established by right nephrectomy combined with two injections of Adriamycin (4 and 2 mg/kg) every 7 days after surgery. This study was carried out in accordance with the Guidelines for the Care and Use of Laboratory Animals of the Research Ethics Committee of the Affiliated Hospital of Nanjing University of Chinese Medicine. The protocol was approved by the Research Ethics Committee of the Affiliated Hospital of Nanjing University of Chinese Medicine.

The rats in the Adriamycin model and TEA groups underwent right nephrectomy under chloral hydrate anesthesia. After surgery, all animals were injected with penicillin at a dose of 200,000 U per day for 3 days. On day 14, the Adriamycin model and TEA groups received Adriamycin (4 mg/kg) by tail vein injection. Meanwhile, rats in the TEA group were given TEA by gavage once a day and rats in the Adriamycin model group received 2 mL of normal saline by gavage daily for 2 weeks. Clinically, the dose of TEA used for a 60-kg human is 5.4 g/day, which converts to 1.5 g/kg/d for rats. On day 21, the second injection of Adriamycin (2 mg/kg) was administered. All rats had free access to water and powdered rat chow. On day 28, all animals were sacrificed and blood, urine, and kidney samples were harvested.

### Reagents and Antibodies

Antibodies against phospho-p38 MAPK (Thr180/Tyr182) (lot no. 4511), p38 MAPK (lot no. 8690), phospho-ERK1/2 (Thr845/Ser967) (lot no. 4370), ERK1/2 (lot no. 4695), phosphor-SAPK/JNK (Thr183/Tyr185) (lot no. 4668), SAPK/JNK antibody (lot no. 9252), β-actin (lot no. 3700), caspase-3 (lot no. 9665), and horseradish peroxidase-conjugated anti-rabbit IgG (lot no. 7074) were obtained from CST Shanghai Biological Reagents Co., Ltd. (Shanghai, China). NLRP3 (lot no. ab214185), caspase 1 (lot no. ab179515), and interleukin-1β (lot no. ab9722) antibodies were purchased from Abcam Trading Co., Ltd. (Shanghai, China). NAC was purchased from Sigma-Aldrich Co., LLC. (Shanghai, China). DMEM/F12 medium and Gibco. FBS were purchased from Thermo Fisher Scientific (Scoresby, Australia). Pen-Strep Amphotericin B Solution was obtained from Biological Industries Israel Beit Haemek Ltd. (Beit Haemek, Israel). Rutin and isoquercitrin standards were purchased from Macklin Chemicals Co., Ltd. (Shanghai, China). Myricetin, hyperoside and quercetin standards were purchased from Aladdin Chemicals Co., Ltd. (Shanghai, China).

### Assessment of Cell Viability With Cell Counting Kit-8

Cell Counting Kit-8 (CCK-8) (Dojindo, Shanghai, China) was used to assess cell viability. Briefly, cells in 96-well plates were exposed to various stimuli according to the manufacturer’s instructions. CCK-8 reagent was added to each well and incubated for 1 h before optical density was measured with a spectrometer at a wavelength of 450 nm. Cell viability was expressed as a percentage of the control.

### Detection of Superoxide and Reactive Oxygen Species Production

The production of both superoxide and ROS was detected using a Total ROS/Superoxide Detection Kit (Enzo Life Sciences, Farmingdale, New York). Briefly, cells were pre-incubated with superoxide detection reagent (orange) or ROS detection reagent (green) in 96-well plates for 1 h and then exposed to various stimulants according to the manufacturer’s instructions. Immunofluorescent images were visualized and captured with an Olympus IX71 inverted fluorescence microscope (Tokyo, Japan) equipped with a standard red and green fluorescence cube. Fluorescence images were analyzed quantitatively with ImageJ software (1.8.0).

### Assessment of Protein Oxidation

Oxidative modification of proteins was evaluated using an OxyBlot Protein Oxidation Detection Kit (EMD Millipore, Billerica, MA) to test for carbonyl groups. Briefly, the protein lysate was prepared in sodium dodecyl sulfate (SDS) lysis buffer (62.5 mM Tris–HCl, 2% SDS, 10% glycerol) together with proteinase inhibitor cocktail (Thermo Fisher Scientific) and 50 mM DL-dithiothreitol. Each protein sample (5 μL) was denatured by adding 5 μL of 12% SDS. Samples were then derivatized by adding 10 μL of 2,4- dinitrophenylhydrazine solution. After incubation at room temperature for 15 min, 7.5 μL of neutralization solution was added to each tube. Samples were then subjected to western blot analysis to detect the carbonyl groups.

### TdT-Mediated dUTP Nick End Labeling (TUNEL) and 4,6-Diamidino-2-Phenylindole (DAPI) Staining of Apoptotic Cells

A TUNEL staining kit (Beyotime Biotechnology, Shanghai, China) was used to assess the presence of apoptotic cells. Briefly, the cells were fixed in 4% paraformaldehyde for 20 min, permeabilized with 0.1% Triton X-100 in phosphate-buffered saline (PBS) for 5 min, and washed with PBS. TUNEL staining solution was added and cells were incubated for 1 h at 37°C. Cells were then washed in PBS. 4,6-diamidino-2- phenylindole (DAPI) staining solution was added to each well and cells were incubated for 5 min in the dark. The staining solution was then removed and the cells were washed in PBS. Finally, cell fluorescence was captured using a fluorescence photometer.

### Flow Cytometry Analysis

Apoptosis was detected using the Annexin V-FITC/propidium iodide (PI) apoptosis assay kit (BD Biosciences, Singapore). Briefly, cells were washed with cold PBS and resuspended in 100 μL of 1× Annexin V binding buffer containing 5 μL of Annexin V-FITC and 5 μL of PI. After incubation for 15 min, 400 μL of 1 × Annexin V binding buffer were added to the cells. The percentage of apoptotic cells was analyzed using a flow cytometer (BD Accuri C6: BD Biosciences, San Jose, CA).

### Transient Transfection of Cells With siRNA

NRK-52E cells were transiently transfected with siRNA specifically targeting NLRP3 (Thermo Fisher Scientific), or a negative control siRNA (All Stars Negative Control siRNA; Qiagen, Hilden, Germany) at a final concentration of 100 nM using HiPerFect transfection reagent (Qiagen). Transfected cells were then either left untreated or exposed to various stimuli.

### Western Blot Analysis

Extracted cellular protein lysates were separated by 7.5, 10, or 12% SDS-polyacrylamide gels and electrotransferred onto polyvinylidene difluoride membranes using protein electrophoresis and blotting equipment (Bio-Rad, Shanghai, China). After blocking with 5% non-fat dried milk in PBS, the membranes were incubated with primary antibody overnight at 4°C. The membranes were washed and probed with horseradish peroxidase-conjugated anti-rabbit or anti-rat IgG for 1 h at room temperature, and the bands were visualized using a Chemidoc Imaging System (Shanghai Tanon Technology Co., Ltd., Tanon 4600SF). β-actin was used as an internal control to confirm the equal loading of proteins. Western blot bands were analyzed quantitatively with ImageJ software (1.8.0).

### Urinary and Serum Sample Analysis

Urinary albumin concentration was identified by Urinary Albumin Assay Kit (Jiancheng Biotechnology, Nanjing, China). The level of serum creatinine was measured by Creatinine Assay Kit (Jiancheng Biotechnology). Serum albumin was evaluated by Albumin Assay Kit (Jiancheng Biotechnology). All procedures were performed according to the manufacturer’s instructions.

### Renal Histomorphometric Analysis

The abdominal cavities of the rats were carefully incised and the left kidney was removed from the renal hilum. The renal cortex and medulla were separated. A small amount of renal cortex at both renal poles was removed and fixed in 10% neutral buffered formalin, dehydrated for 24 h, and embedded in paraffin. The samples were cut into 3-μm-thick slices and stained with Hematoxylin and Eosin staining reagent and Masson staining kit. The renal tubulointerstitial tissues and glomeruli were observed under a light microscope.

### Immunohistochemistry

The slides were washed with PBS after deparaffinization and antigen retrieval. After blocking with 3% bovine serum albumin at room temperature for 30 min, the samples were incubated with a primary antibody overnight at 4°C. Slides were then washed with PBS and incubated with a secondary antibody at room temperature for 50 min. After drying, freshly prepared 3,3’-diaminobenzidine chromogenic reagent was added to the marked tissue. Slides were observed under a microscope until the nuclei turned yellow-brown, and then counterstained with hematoxylin staining solution. Finally, the samples were dehydrated, cleared in xylene for 5 min and mounted with resin mounting medium. The tissues were observed under light microscopy.

### Statistical Analysis

Values are expressed as the means ± standard deviation. Comparisons of two populations were performed by the Student’s *t*-test. For multiple comparisons, one-way analysis of variance followed by Dunnett’s test was employed. All analyses were performed using SPSS Statistics 23.0 software (IBM Corp., Armonk, NY). A *P* value of less than 0.05 was considered to indicate a statistically significant difference.

## Results

### Adriamycin Triggers Renal Tubular Cell Injury

**FIGURE 2:**
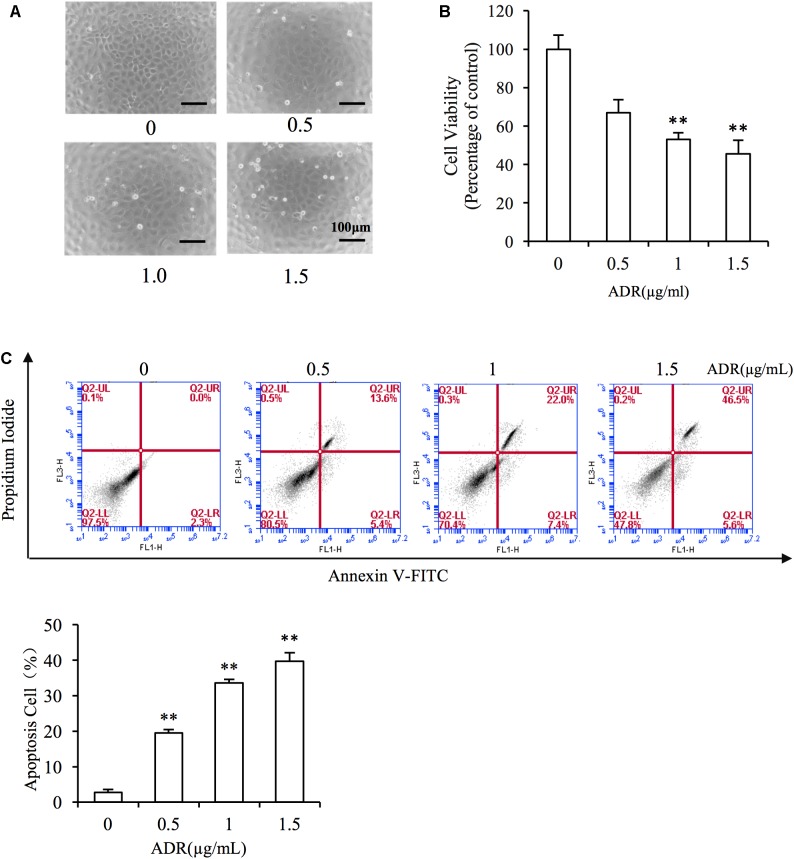
Adriamycin elicited renal tubular cell injury. **(A)** Role of Adriamycin (ADR) in morphological changes. NRK-52E cells were treated with different concentrations of ADR (0, 0.5, 1.0, 1.5 μg/mL) for 24 h. Cell morphology was analyzed using phase-contrast microscopy (magnification, ×100). **(B)** Effects of ADR on cell viability. NRK-52E cells in 96-well plates were exposed to the different concentrations (0, 0.5, 1.0, 1.5 μg/mL of ADR for 24 h. Cell viability was evaluated using a CCK-8 assay. Data are expressed as the percentages of living cells versus the control (Ctrl) (means ± SD, *n* = 5). ^∗∗^*P* < 0.01 versus Ctrl. **(C)** The effects of ADR in flow cytometry assay. NRK-52E cells in 6-well plates were treated with different concentrations of ADR (0, 0.5, 1.0, 1.5 μg/mL) for 24 h and the apoptotic cells were evaluated by flow cytometry to detect labeled Annexin V-FITC/PI. Flow cytometry analysis of apoptosis is shown at the bottom. ^∗∗^*P* < 0.01 versus Ctrl.

First, we evaluated the cytotoxicity of Adriamycin to NRK-52E renal tubular epithelial cells. As shown in Figure 2A,B, incubation with Adriamycin induced dose-dependent detachment of cells from the bottom of the culture dish and loss of cell viability. Our previous study suggested that Adriamycin triggers oxidative stress-mediated apoptosis in podocytes (Gao et al., 2014). Similarly, Adriamycin evoked apoptosis in a concentration-dependent manner as revealed by the increased signal in the upper-right quadrant in flow cytometry analysis (Figure 2C). These results indicated that Adriamycin triggered renal tubular cell injury.

### TEA Ameliorates Adriamycin-Induced Renal Tubular Cell Injury

**FIGURE 3:**
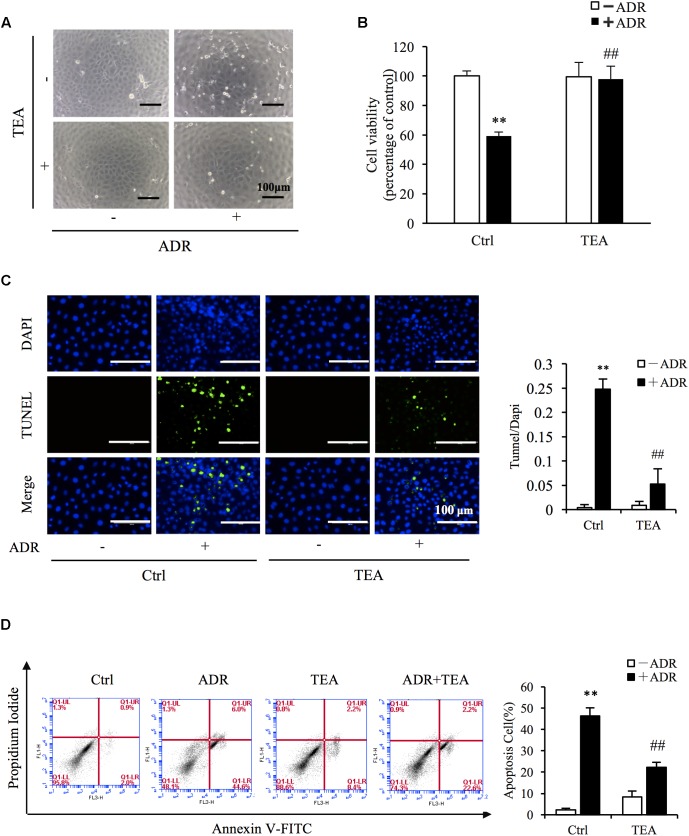
TEA ameliorates ADR-elicited tubular cell injury. **(A)** Role of TEA in ADR-induced morphological changes. NRK-52E cells were pretreated with TEA (100 μg/mL) for 1 h and challenged with ADR for 24 h. Cell morphology was analyzed using phase-contrast microscopy (magnification, ×100). **(B)** Effects of TEA on cell viability. NRK-52E cells in 96-well plates were pretreated with TEA (100 μg/mL) for 1 h and challenged with ADR for 24 h. Cell viability was evaluated by the CCK-8 assay. Data are expressed as the percentages of living cells versus Ctrl (means ± SD, *n* = 5). ^∗∗^*P* < 0.01 versus Ctrl. ##*P* < 0.01 versus ADR in Ctrl. **(C)** Apoptosis staining of NRK-52E cells. NRK-52E in 48-well plates were pretreated with TEA (100 μg/mL) for 1 h and challenged with ADR for another 24 h. Apoptotic cells were evaluated by TUNEL and DAPI staining. Data on the right are expressed as the percentages of dead cells compared with the Ctrl (means ± SD, *n* = 5; ^∗∗^*P* < 0.01 versus Ctrl. ##*P* < 0.01 versus ADR in Ctrl). **(D)** The effects of TEA in flow cytometry assay. NRK-52E cells in 6-well plates were pretreated with TEA (100 μg/mL) for 1 h and challenged with ADR for another 24 h. The apoptotic cells were detected by flow cytometry to detect labeled Annexin V-FITC/PI. Flow cytometry analysis of apoptosis is shown on the right. ^∗∗^*P* < 0.01 versus Ctrl; ##*P* < 0.01 versus ADR in Ctrl.

To evaluate the role of TEA in Adriamycin-induced renal cell injury, we pretreated the cells with TEA. As observed, TEA attenuated Adriamycin-induced cellular morphological changes and loss of cell viability (Figure 3A,B). Furthermore, TEA mitigated Adriamycin-elicited apoptosis as verified by light TUNEL staining of apoptotic cells in TEA-treated cells (Figure 3C). This anti-apoptotic role was further validated by flow cytometry analysis (Figure 3D). These data showed that TEA indeed ameliorated Adriamycin-elicited cell impairment.

### Oxidative Stress Underlies the Role of TEA Against the Cytotoxicity of Adriamycin

**FIGURE 4:**
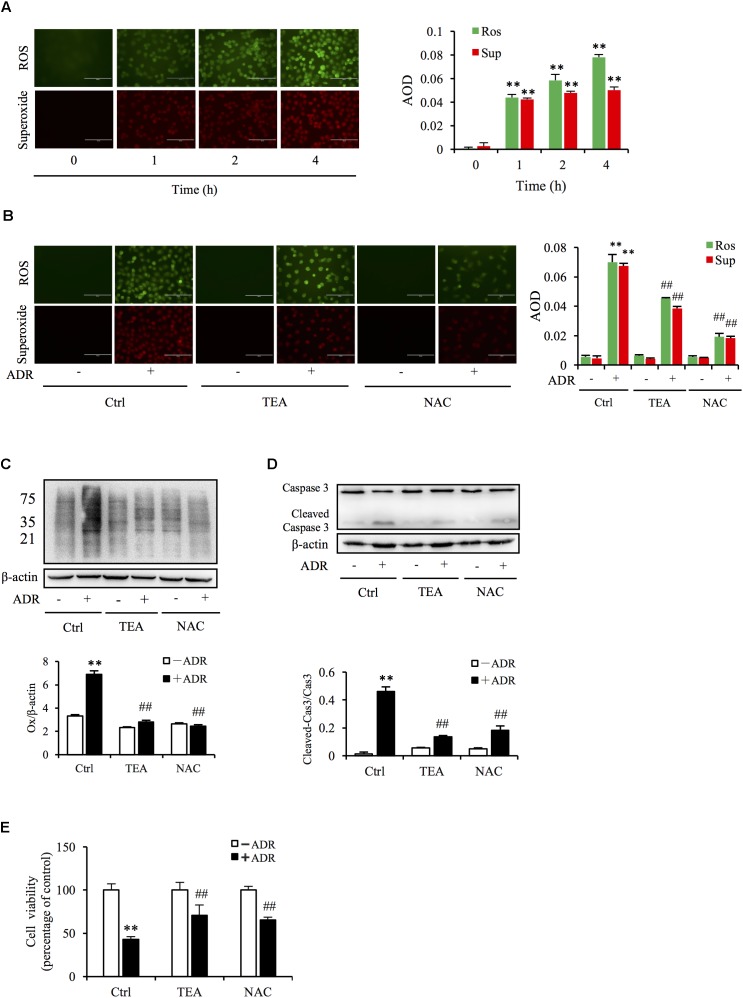
Oxidative stress underlies ADR-induced cell injury. **(A)** Effects of ADR on superoxide and ROS production. NRK-52E in 96-well plates were incubated with ADR for different periods and loaded with superoxide and ROS detection reagents for 1 h. The cells were analyzed by fluorescence microscopy (magnification, ×400). Quantitative measurements of the fluorescence intensities were conducted using ImageJ software. Data are expressed as the relative intensities against zero point control (means ± SD, *n* = 3; ^∗∗^*P* < 0.01 versus Ctrl). **(B)** Effects of TEA and NAC on superoxide and ROS production triggered by ADR. NRK-52E in 96-well plates were pretreated with TEA (100 μg/mL) or NAC (5 mM) for 1 h, challenged with ADR for 3 h, then loaded with superoxide and ROS detection reagent for another 1 h. The cells were subsequently analyzed by fluorescence microscopy (magnification, ×400). Quantitative measurements of the fluorescence intensities are shown at the bottom (means ± SD, *n* = 3; ^∗∗^ *P* < 0.01 versus Ctrl; ##*P* < 0.01 versus ADR in Ctrl). **(C)** Role of TEA and NAC on oxidative modification of proteins induced by ADR. NRK-52E cells were pretreated with TEA (100 μg/mL) and NAC (5 mM) in 12-well plates for 1 h and incubated with ADR for another 4 h. Thereafter, cellular lysates were analyzed by OxyBlot Protein Oxidation Detection Kit and immunodetection of carbonyl groups. β-actin was used as an internal control. Densitometric analysis of protein oxidation is shown at the bottom (means ± SD, *n* = 3; ^∗∗^*P* < 0.01 versus Ctrl; ##*P* < 0.01 versus ADR in Ctrl). **(D)** Effects of TEA and NAC on caspase 3 cleavage induced by ADR. NRK-52E cells in 12-well plates were pretreated with TEA (100 μg/mL) and NAC (5 mM) for 1 h and incubated with ADR for 24 h. Cellular lysates were analyzed by western blots targeting caspase 3 and cleaved-caspase 3. Densitometric analysis of cleaved-caspase 3 is shown at the bottom (means ± SD, *n* = 3; ^∗∗^ *P* < 0.01 versus Ctrl; ## *P* < 0.01 versus ADR in Ctrl). **(E)** Effects of TEA and NAC on cell viability. NRK-52E cells in 96-well plates were pretreated with TEA or NAC for 1 h and challenged with ADR for 24 h. Cell viability was evaluated by CCK-8 assay. Data are expressed as percentages of dead cells versus Ctrl (means ± SD, *n* = 5). *p* < 0.01 versus Ctrl; ## *P* < 0.01 versus ADR in Ctrl.

Given that superoxide and ROS are involved in Adriamycin-triggered oxidative injury, we tested the production of ROS via immunofluorescence probe. As shown in Figure 4A, Adriamycin increased the generation of superoxide (red) and ROS (green) in a time-dependent manner. Interestingly, TEA slightly suppressed Adriamycin-induced overproduction of these oxidants compared to N-acetylcysteine (NAC), a well-known antioxidant (Figure 4B). Oxidative stress results in the oxidative damage of macromolecules including proteins, nucleoids, and lipids. Thus, we next compared the role of TEA with NAC on protein oxidative modifications. Protein carbonyl level is an index of oxidative protein injury, which can be detected by the DNPH reaction via western blot analysis of the modified protein. As shown in Figure 4C, consistent with the role of NAC, TEA relieved Adriamycin-induced production of these oxidants. Furthermore, TEA and NAC suppressed Adriamycin-elicited apoptosis as shown by the change in the cleaved caspase-3 band in western blot analysis (Figure 4D) and cell viability (Figure 4E). These outcomes suggested that the protective role of TEA against the toxicity of Adriamycin was mediated, at least in part, by antioxidative stress independently of the inhibition of ROS overproduction.

### TEA Protects Renal Tubules Against Toxicity of Adriamycin by Suppression of Oxidative Stress-Mediated ERK1/2 Signaling

**FIGURE 5:**
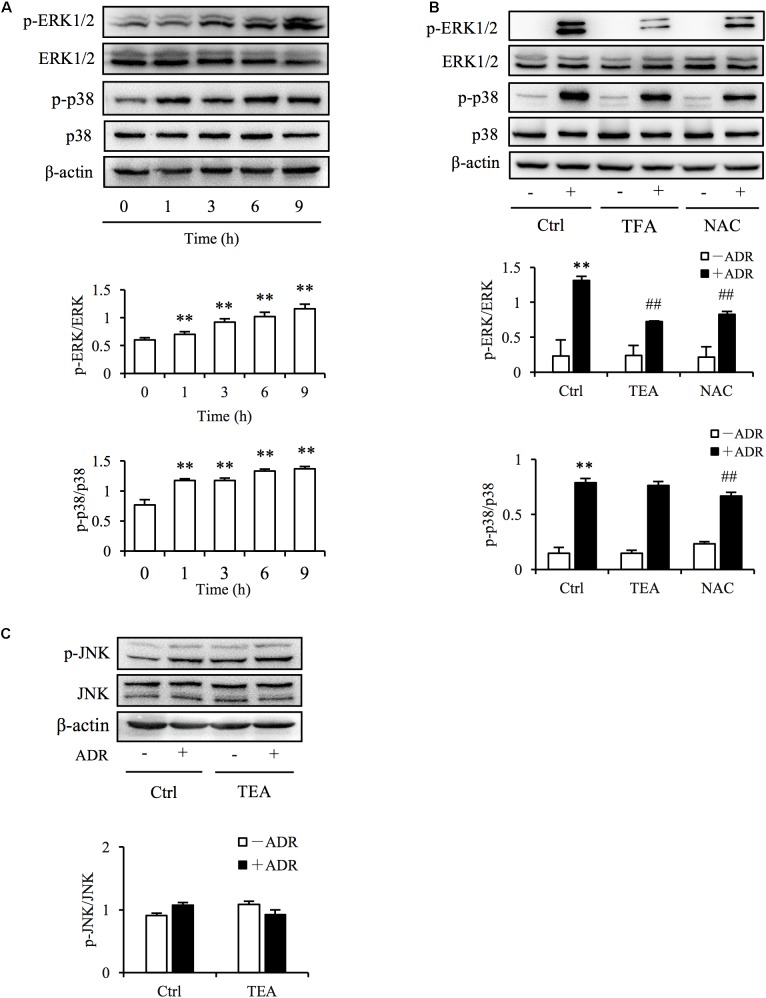
TEA regulates ERK1/2 signaling. **(A)** Induction of p38 and ERK1/2 phosphorylation by ADR. NRK-52E cells in 12-well plates were incubated with ADR for 1, 3, 6, and 12 h Cellular lysates were analyzed by western blot for phosphorylated p38 and ERK1/2. Statistical analyses of phosphorylated ERK1/2 and p38 are shown at the bottom (means ± SD, *n* = 3; ^∗∗^*P* < 0.01 versus Ctrl). **(B)** Effects of TEA and NAC on ERK1/2 and p38 phosphorylation induced by ADR. NRK-52E cells in 12-well plates were pretreated with TEA (100 μg/mL) and NAC (5 mM) for 1 h and incubated with ADR for another 6 h. Lysates were analyzed by western blots targeting phosphorylated p38 and ERK1/2. Statistical analysis of phosphorylated ERK1/2 and p38 are shown at the bottom (means ± SD, *n* = 3; ^∗∗^*P* < 0.01 versus Ctrl; ## *P* < 0.01 versus ADR in Ctrl). **(C)** Effects of TEA on JNK phosphorylation induced by ADR. NRK-52E cells in 12-well plates were pretreated with TEA (100 μg/mL) and NAC (5 mM) for 1 h and challenged with ADR for another 6 h. Lysates were analyzed by western blot targeting phosphorylated JNK. Statistical analyses of phosphorylated JNKs are shown at the bottom.

The definition of oxidative stress has changed from the emphasis on an imbalance of pro-oxidants and antioxidants to focusing on the disruption of specific redox signaling and control pathways (Jones, 2006). Increased ROS level is always associated with the activation of oxidative stress-sensitive kinase and redox signaling. Mitogen-activated protein kinase (MAPK) signaling is sensitive to stressors including oxidative, chemical, and physical stress. To determine if TEA influenced ROS-mediated signaling, we observed extracellular-signal–regulated kinases 1 and 2 (ERK1/2), p38, and c-Jun N-terminal kinase (JNK) signaling transduction. As shown in Figure 5A, Adriamycin potently activated ERK1/2 and p38 kinases. Intriguingly, TEA prohibited ERK1/2 phosphorylation more than did p38 (Figure 5B). Moreover, TEA did not influence signaling of JNKs triggered by Adriamycin (Figure 5C). TEA also suppressed hydrogen peroxide (H_2_O_2_)-induced activation of ERK1/2 (Supplementary Figure 1A). H_2_O_2_ is a classic ROS inducer (Supplementary Figure 1B). Hence, it is possible that ERK1/2 signaling transduction was the specific target of TEA in Adriamycin-induced renal tubular cell injury.

### Adriamycin Activated the NLRP3 Inflammasome by Oxidative Stress-Mediated ERK1/2 Signaling

**FIGURE 6:**
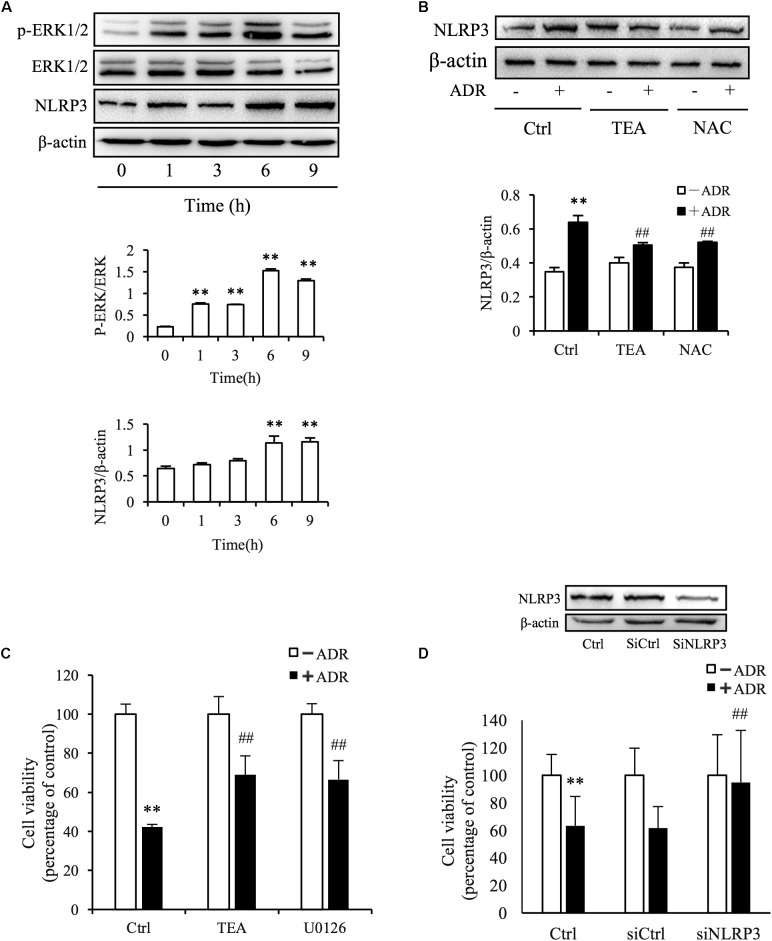
ADR induces oxidative stress-mediated NLRP3 inflammasome activation. **(A)** Induction of ERK1/2 phosphorylation and NLRP3 protein levels by ADR. NRK-52E cells in 12-well plates were incubated with ADR for 1, 3, 6, and 9 h, respectively. Statistical analyses of phosphorylated ERK1/2 and NLRP3 are shown at the bottom (means ± SD, *n* = 3; ^∗∗^*P* < 0.01 versus Ctrl). **(B)** Effects of TEA and NAC on NLRP3 induced by ADR. NRK-52E cells in 12-well plates were pretreated with TEA or NAC for 1 h and incubated with ADR for 9 h. Statistical analyses of NLRP3 are shown at the bottom (means ± SD, *n* = 3; ^∗∗^*P* < 0.01 versus Ctrl; ##*P* < 0.01 versus ADR in Ctrl). **(C)** Effects of TEA and U0126 on cell viability. NRK-52E cells in 96-well plates were pretreated with TEA (100 μg/mL) or U0126 (20 μM) for 1 h and challenged with ADR for 24 h. Cell viability was evaluated by CCK-8 assay. Data are expressed as percentages of living cells versus control (means ± SD, *n* = 5). ^∗∗^*P* < 0.01 versus control; ## *P* < 0.01 versus ADR in Ctrl. **(D)** Effects of NLRP3 siRNA on ADR-induced cell injury. NRK-52E cells were transfected with either NLRP3 siRNA or control siRNA for 24 h. The transfected cells were incubated with ADR for 24 h. Cell viability was evaluated using a CCK-8 assay. Data are expressed as the percentages of living cells compared with the siRNA controls (means ± SD, *n* = 4; ^∗∗^*P* < 0.01 versus siRNA control; ## *P* < 0.01 versus ADR in Ctrl).

**FIGURE 7:**
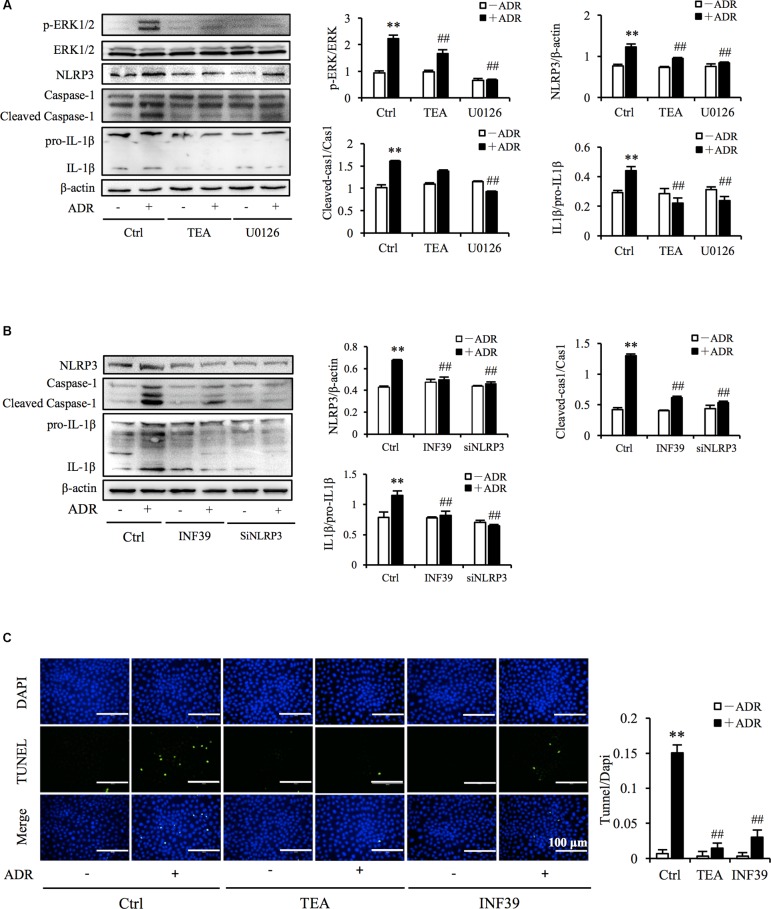
TEA regulates ERK1/2 mediated NLRP3 inflammasome activation. **(A,B)** Effect of TEA, U0126, INF9, and NLRP3 siRNA on ERK1/2 and NLRP3 inflammasome activation. NRK-52E cells were transfected with either NLRP3 siRNA or control siRNA for 24 h. Thereafter transfected cells were incubated with ADR for 24 h. Wild type NRK-52E cells in 12-well plates were pretreated with TEA (100 μg/mL), U0126 (20 μM), or INF39 (100 μM) for 1 h and challenged with ADR for 24 h. Statistical analysis of targeted proteins is shown at the bottom (means ± SD, *n* = 3; ^∗∗^*P* < 0.01 versus Ctrl; ##*P* < 0.01 versus ADR in Ctrl). **(C)** Apoptosis staining of NRK-52E cells. NRK-52E in 48-well plates were pretreated with TEA (100 μg/mL) and INF39 for 1 h and challenged with ADR for another 24 h. Apoptotic cells were evaluated by TUNEL and DAPI staining. Data at the bottom are expressed as the percentages of dead cells compared with the Ctrl (means ± SD, *n* = 5; ^∗∗^*P* < 0.01 versus Ctrl. ##*P* < 0.01 versus ADR in Ctrl).

It has been reported that ROS and Adriamycin can activate inflammasomes (Nicholas et al., 2011; Sauter et al., 2011). NLRP3 belongs to the NLR family of pattern recognition receptors and is one of the most widely studied inflammasomes. NLRP3 senses cellular stress signals such as oxidative stress. Thus, NLRP3 may be involved in Adriamycin-induced renal tubular oxidative injury. As shown in Figure 6A, Adriamycin phosphorylated ERK1/2 as soon as 1 h after treatment and was followed by an increase in NLRP3 protein level after 6 h. Furthermore, upregulation of NLRP3 protein triggered by Adriamycin was suppressed by TEA and NAC (Figure 6B). Moreover, similar to the role of TEA, the inhibition of ERK1/2 by the pharmacological inhibitor U0126 alleviated the loss of cellular viability caused by Adriamycin (Figure 6C). Downregulation of NLRP3 by siRNA ameliorated the loss of cellular viability, which further confirmed the role of NLRP3 (Figure 6D). INF39 is one of the inhibitors of NLRP3. TEA, U0126, and INF39 all inhibited NLRP3, caspase-1 activation, and Il-1β maturation (Figure 7A,B). TEA and NLRP3 both inhibited the attenuation of Adriamycin-triggered apoptosis, as shown in TUNEL apoptosis staining (Figure 7C). These outcomes showed that TEA suppressed ERK1/2-mediated NLRP3 inflammasome activation.

### TEA Attenuates Proteinuria and Improves Hypoalbuminemia in Rats With Adriamycin Nephropathy

**FIGURE 8:**
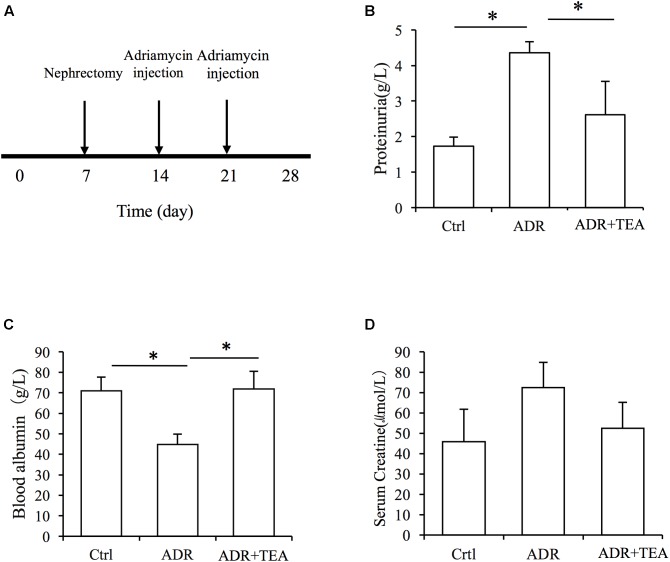
TEA attenuates proteinuria and improves hypoproteinemia in rats with Adriamycin nephropathy. **(A)** Experimental procedure. The ADR and TEA groups underwent right nephrectomy on day 7 and ADR was injected through the tail vein on days 14 and 21. The rats were sacrificed on day 28. TEA was given by gavage once a day at a dosage of 1.5 g/kg/d in the treatment group. Rats in the model group received 2 mL normal saline by gavage daily from day 14. Comparisons of urinary protein **(B)**, blood albumin **(C)**, and serum creatinine **(D)** levels among the 3 groups (means ± SD) (^∗^*P* < 0.05 between groups).

Adriamycin nephropathy is characterized by podocyte and tubular cell injury. Proteinuria is the result of both podocyte and tubular cell damage. We established AN in rats (Figure 8A). After injection of Adriamycin, proteinuria increased but administration of TEA significantly reduced proteinuria (Figure 8B). Moreover, there was a remarkable decrease in serum albumin in AN rats, and TEA improved hypoalbuminemia (Figure 8C). However, there was no significant change in serum creatinine levels after Adriamycin injections (Figure 8D).

### TEA Improves Renal Tissue Lesions in Rats With Adriamycin Nephropathy

**FIGURE 9:**
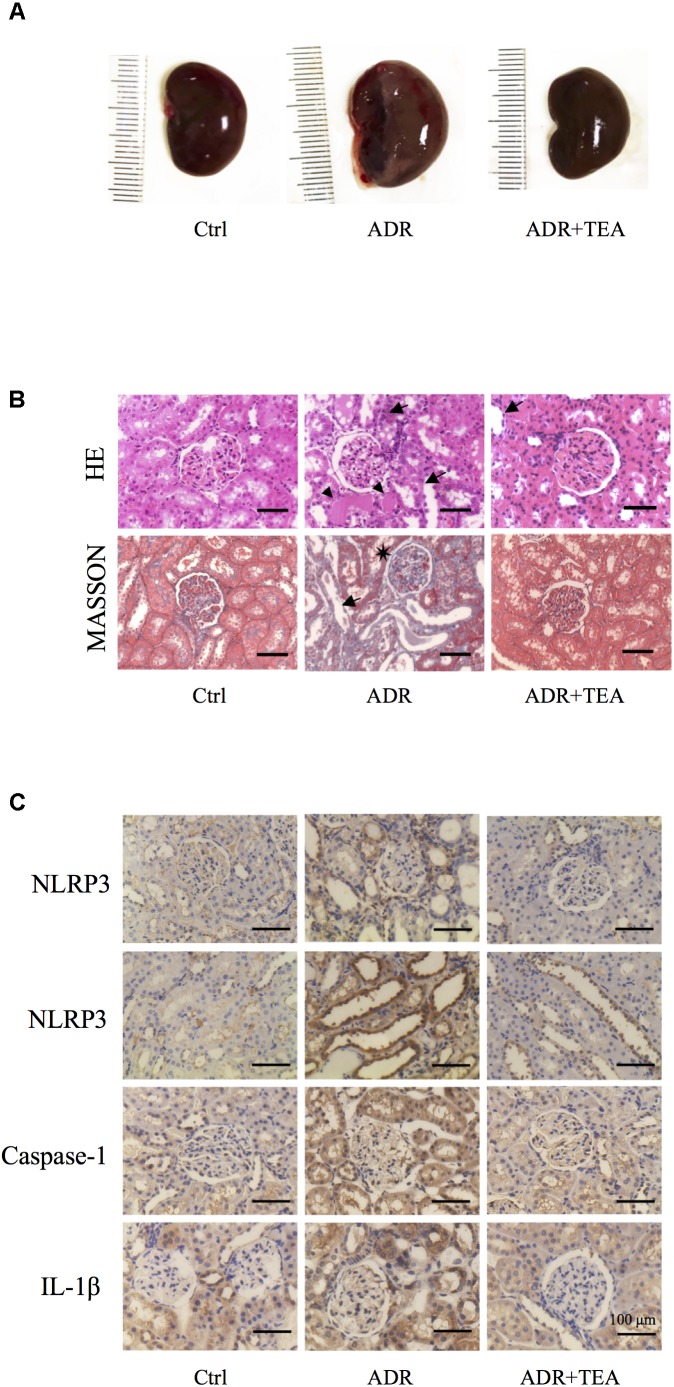
TEA improves renal pathology in rats with Adriamycin nephropathy. **(A)** Macroscopic morphology of the kidneys. **(B)** Hematoxylin and Eosin staining: Renal tubular epithelial cells in the normal group were in alignment and had normal shapes. In the ADR group, the renal tubular epithelial cells were shed, and some of the bare membranes were visible, while some of them had regenerated (arrows). Protein casts are indicted as (Δ). Granular degeneration can be seen in proximal tubular cells. Renal tubular epithelial cells were detached (arrows) in the treatment group, which was at a lower level compared with the ADR group. Masson staining: Capillary vasospasm and buccal segmental adhesion (^∗^) were detected in the Adriamycin group. Renal tubular epithelial cells were detached, and bare membranes (arrow) were visible. There was no obvious fibrosis in the renal interstitium. Scale bar: 100 μm. **(C)** Representative images of sections assayed by immunohistology using NLRP3, Caspase 1, and IL-1β antibody. Scale bar: 100 μm.

Gross examination showed that kidneys in AN were enlarged compared with controls, and that this was attenuated by TEA (Figure 9A). As shown in Figure 9B,C, tubule lesions were histologically visible to varying degrees with no notable interstitial infiltrate of cells and fibrosis. We found varying numbers of degenerating and regenerating tubular epithelial cells and loss of individual lining cells. Vacuoles were seen in some tubular cells, and the lining cells were flattened. Brush border staining was reduced or absent. Partially desquamated cells and protein casts were observed in dilated tubules. TEA ameliorated the lesions mentioned above. As shown in Figure 9C, Adriamycin increased the expression of NLRP3, caspase-1, and IL-1β in renal tubular cells. TEA suppressed the expression levels of NLRP3 inflammasome proteins. Interestingly, NLRP3 was mainly expressed in the tubules rather than in glomeruli (first line of images in Figure 9C). These results implied that TEA attenuated Adriamycin-induced renal pathological changes and inhibited NLRP3 inflammasomes in the tubules.

## Discussion

**FIGURE 10:**
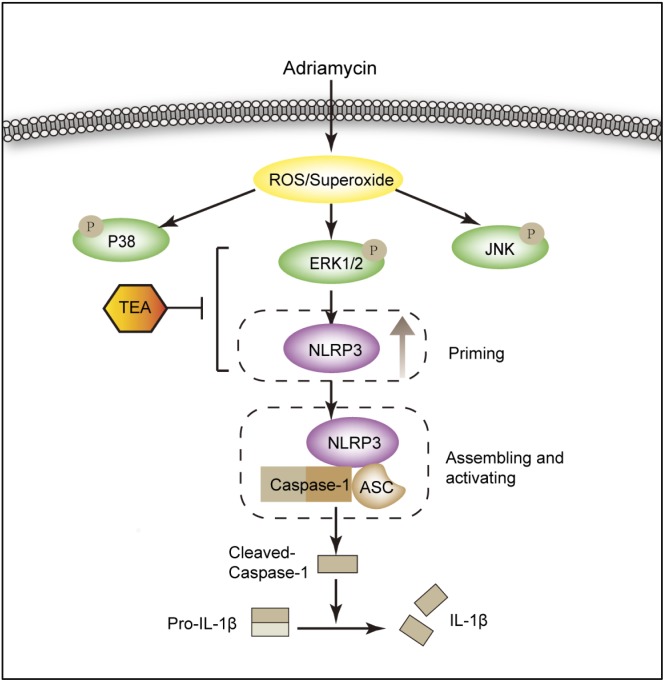
Diagram of the mechanism by which TEA regulates the NLRP3 inflammasome. Adriamycin induces oxidative stress by increasing the overproduction of ROS. ERK1/2 kinases mediate ROS-triggered activation of the NLRP3 inflammasome. TEA reduces NLPR3 via suppression of ERK1/2 kinases and subsequently inhibits the NLRP3 inflammasome activation induced by Adriamycin. Thus, TEA ameliorates Adriamycin-induced renal tubule injury via suppression of ROS-ERK1/2-mediated NLRP3 priming process.

For the first time, we demonstrated that TEA attenuated Adriamycin-induced renal tubular cell injury. This protective role of TEA is ascribed to the inhibition of ROS-ERK1/2-NLRP3 inflammasome activation (Figure 10). Our data suggest a novel mechanism of TEA that might protect renal tubules and emphasizes the crucial role of ROS-ERK1/2-NLRP3 inflammasome signal during kidney injury.

Among the major compounds in TEA, hyperoside, and isoquercitrins are classified as flavonoid glycosides, whereas myricetin and quercetin are classified as flavonoid aglycones (Guo et al., 2010; Guo et al., 2015). Similar results to those following TEA were not obtained with quercetin or hyperoside alone at high concentrations from *A. manihot*, in the Adriamycin-induced renal cell injury model *in vitro*. Several studies have reported the proteinuria-lowering effects of *A*. *manihot*. Oral administration of TEA (200 mg/kg per day) for 24 weeks inhibited the overexpression of caspases 3 and 8, and ameliorated urinary albumin excretion, glomerular hyperfiltration, and podocyte apoptosis at an early stage in a diabetic nephropathy rat model (Zhou et al., 2012). In our previous study *in vivo*, an extract of *A. manihot* (Huang Kui capsule), suppressed inflammation, glomerulosclerosis, mesangial cell proliferation, extracellular matrix and collagen deposition, α-smooth muscle actin, and collagen type I expression in an AN rat model. Thus, anti-inflammatory effects were thought to be the most important mechanism that led to the reduction in proteinuria (Tu et al., 2013; Mao et al., 2015). In addition to *in vivo* studies, some clinical trials have also confirmed the proteinuria-lowering role of TEA (Zhang et al., 2014). We lack a detailed understanding of the mechanisms involved in kidney disease because of the versatile pharmacological actions of *A. manihot*.

Traditionally, more attention has been paid to the podocyte and maintenance of the integrity of the glomerular bottom membrane (GBM) in proteinuria reduction therapy. Many studies have raised numerous concerns about the role of renal tubular cells in protein-related kidney disease (Tang et al., 2011). Renal epithelial cells are sensitive to all kinds of stimuli including drugs, toxins, and stress. Tubular loss is one of the issues of AN. In Adriamycin nephrosis, tubular injury is generally considered to be a direct toxic effect of Adriamycin and secondary to glomerular injury and proteinuria. Oxidative stress, inflammation, apoptosis, and DNA damage are thought to underlie the toxic effects of Adriamycin. Among them, oxidative stress is perceived to be one of the most crucial mechanisms (Morgan et al., 1998; Yang et al., 2009).

Oxidative stress is a disruption of redox signaling and control rather than a disturbance in the prooxidant-antioxidant balance in favor of the former (Jones, 2006). Thus, oxidative stress emphasizes the disruption of specific redox signaling and control pathways. ERK pathway is the first signaling cassette of MAPK signaling pathways to be discovered and is composed of ERK1/2, p38 MAPK, JNK1-3, and ERK5. Oxidative stress is known to induce the activation of ERK1/2 via upstream factors such as calcium channels, receptor tyrosine kinases, RAS, and Src (McCubrey et al., 2006). ERK1/2 signaling participates in key physiological processes that control cell proliferation, differentiation, survival, and death (Ramos, 2008).

In the current study, TEA did not suppress the generation of ROS significantly compared with NAC, and several considerations prompted us to speculate on the involvement of antioxidative stress. First, Adriamycin triggered the overproduction of ROS and activated ERK1/2 signaling. Second, TEA suppressed protein oxidation and ERK1/2 activation in a manner similar to NAC. Third, TEA attenuated cell injury triggered by Adriamycin, consistent with NAC. Therefore, anti-oxidative stress underlies the process by which TEA preserves renal tubular cells. Moreover, the greater inhibitory effect of TEA on ERK1/2 rather than p38 activated by Adriamycin indicated that TEA specifically regulated ROS-ERK1/2 signaling to control the cells in response to extracellular and intracellular stimuli.

In response to the detection of pathogen-associated molecular patterns (PAMPs) or danger associated molecular patterns (DAMPs), inflammasomes assemble to activate caspase-1, which cleaves the pro-inflammatory cytokines interleukin-1β (IL-1β) and IL-18 from their pro-forms into their secreted mature forms. Inflammasomes have been implicated in a broad range of common inflammatory diseases such as sepsis, lung injury, diabetes mellitus, and rheumatoid arthritis, as well as rarer inherited disorders such as familial Mediterranean fever and assorted cryopyrin-associated auto-inflammatory diseases (Latz et al., 2013). Inflammasomes are categorized according to their sensor proteins: NLRP1, NLRP3, NLRC4 (NOD-like receptor family CARD domain-containing protein 4), AIM2 (Absent in melanoma-2), and pyrin. The NLRP3 inflammasome is the most widely studied inflammasome and responds to a broad range of PAMP/DAMPs. It forms a complex with the adaptor molecule, an apoptosis-associated speck-forming complex containing CARD (ASC). The NLRP3:ASC complex oligomerizes and binds the enzyme caspase 1, leading to caspase 1 activation and the conversion of pro-IL-1β and -18 into their mature forms, thus promoting tissue influx of leukocytes and creating conditions favorable to inflammation. Accumulating data suggests that the inflammasome is activated in patients with CKD as well as in animal models of this condition (Martinon and Tschopp, 2007; Vilaysane et al., 2010; Anders and Muruve, 2011; Zhuang et al., 2014). Recently, it has been reported that suppression of the NLRP3 inflammasome attenuates renal injury (Foresto-Neto et al., 2018).

NLRP3 inflammasomes can be activated by a wide range of physical and chemical stimuli, including exogenous microbial stimuli, environmental large inorganic crystalline structure, and endogenous danger signals. To date, overproduction of ROS is considered to be the most prominent trigger (Bauernfeind et al., 2011). ROS are ancient and highly conserved danger signals, and elevated ROS production is observed upon treatment with many NLRP3 activators tested to date (Cruz et al., 2007; Dostert et al., 2008). In the present study, Adriamycin triggered the overproduction of ROS and superoxide, thus activating the NLRP3 inflammasome – but how does it do so?

NLRP3 inflammasome activation requires two distinct steps: priming activation. Priming involves a kinase-mediated synthesis of pro-IL-1β and the upregulation of NLRP3. The second signal induces the assembly and activation of the inflammasome. It is well established that oxidative stress activates the MAPK family including ERK1/2, p38 MAPK, and JNK signaling (McCubrey et al., 2006). According to our results, the role of TEA is mediated by ERK1/2 signaling. It has been reported that ERK1-mediated post-translational modifications give license to the NLRP3 inflammasome to respond to the second signal ATP by inducing posttranslational events. These data support the concept that ERK1/2 signaling is a central event in inflammasome priming (Ghonime et al., 2014). From our data, Adriamycin activated ROS-mediated ERK1/2 and led to upregulation of the NLRP3 protein level. TEA abolished the Adriamycin-induced increase in NLRP3. Collectively, we confirmed that TEA suppressed NLRP3 inflammasome activation in the priming process via inhibition of ERK1/2 signaling. However, how TEA influenced the ERK1/2 still needs further study. Additionally, ERK1/2 is the upstream of NLRP3 and suppression of ERK1/2 results in reduced protein level of NLRP3. Whether upregulating of ERK1/2 could lead to increased NLRP3 protein or activation of inflammasome? In future study, we will confirm the detailed relationship between ERK1/2 and NLRP3.

Taken together, our results revealed a key role of the ROS-ERK1/2-NLRP3 inflammasome in renal tubular cell injury. Our outcomes suggest a novel drug that could regulate the NLRP3 inflammasome. Therapeutic strategies targeting tubular epithelial cells hold promise for the treatment of some kidney diseases.

## Ethics Statement

This study was carried out in accordance with the Guidelines for Care and Use of Laboratory Animals of the Research Ethics Committee of Affiliated Hospital of Nanjing University of Chinese Medicine. The protocol was approved by the Research Ethics Committee of Affiliated Hospital of Nanjing University of Chinese Medicine.

## Author Contributions

KG and WS designed the research. WL, PX, MS, YZ, BHL, JJZ, and YYZ performed the research. WL and KG analyzed the data. WWZ, WL, and KG analyzed the pathology. WS, LZ, WMH, ECZ, and MJS participated in intellectual discussions. WL and KG wrote the manuscript. All authors approved the final edited version.

## Conflict of Interest Statement

The authors declare that the research was conducted in the absence of any commercial or financial relationships that could be construed as a potential conflict of interest.
