# Supplementary figures and images for: Total Extracts of Abelmoschus manihot L. Attenuates Adriamycin-Induced Renal Tubule Injury via Suppression of ROS-ERK1/2-Mediated NLRP3 Inflammasome Activation
Source: Front Pharmacol. 2019 May 28;10:567. doi: 10.3389/fphar.2019.00567 (PMC6548014; doi:10.3389/fphar.2019.00567)

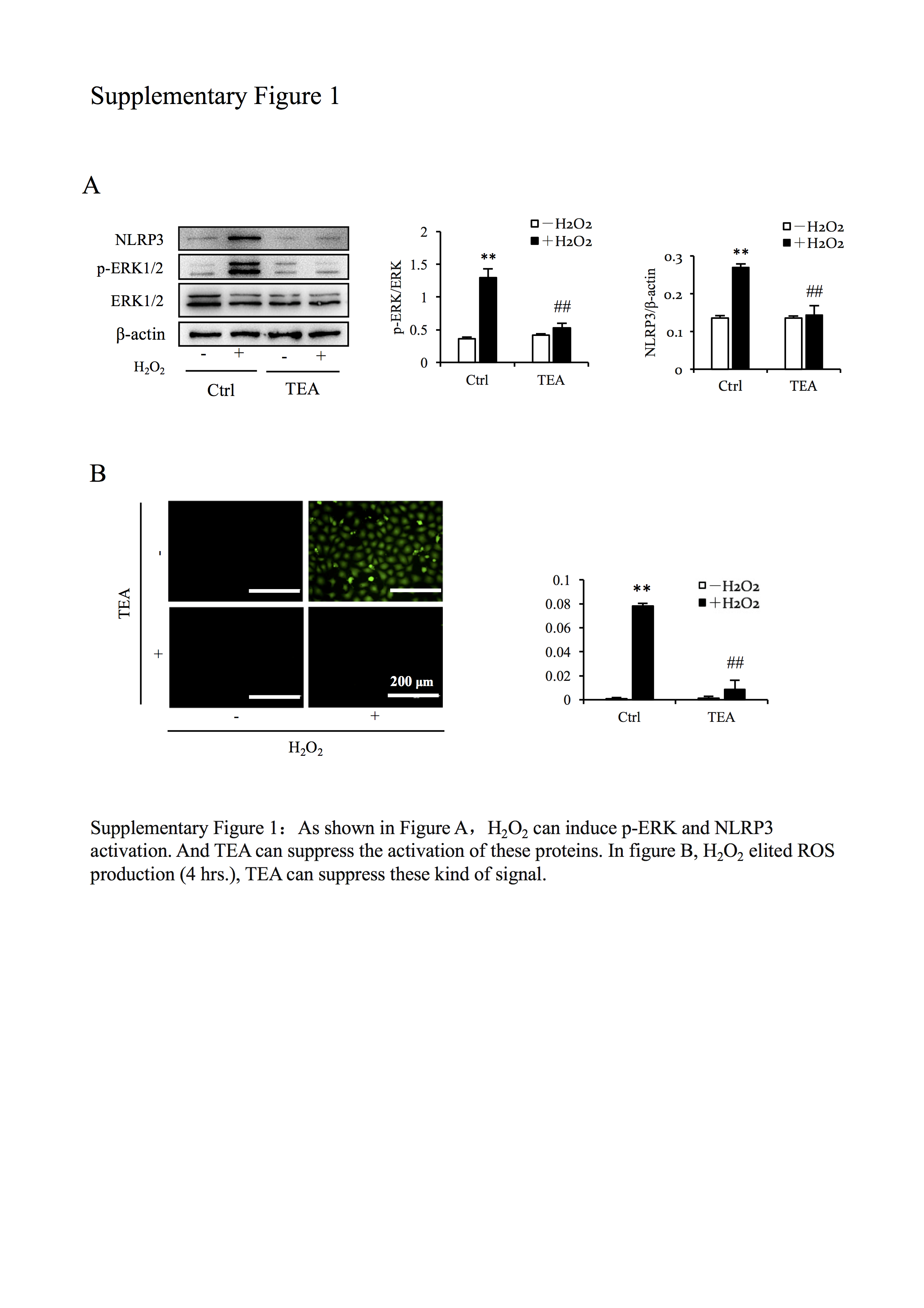

Supplement: Supplementary file 1 [file Image_1.tiff]
